# Co-Polymers based on Poly(1,4-butylene 2,5-furandicarboxylate) and Poly(propylene oxide) with Tuneable Thermal Properties: Synthesis and Characterization

**DOI:** 10.3390/ma12020328

**Published:** 2019-01-21

**Authors:** Marina Matos, Andreia F. Sousa, Patrícia V. Mendonça, Armando J. D. Silvestre

**Affiliations:** 1 CICECO and Department of Chemistry, University of Aveiro, 3810-193 Aveiro, Portugal; marina.matos@ua.pt (M.M.); armsil@ua.pt (A.J.D.S.); 2 CEMMPRE, Department of Chemical Engineering, University of Coimbra, Rua Sílvio Lima, Pólo II, 3030-790 Coimbra, Portugal; patmend@gmail.com

**Keywords:** 2,5-Furandicarboxylic acid, poly(propylene oxide), poly(ester-ether) co-polymers, tuneable thermal properties

## Abstract

Poly(ether ester)s (PEEs) represent a promising class of segmented co-polymers, nevertheless the synthesis of PEEs based on renewable 2,5-furandicarboxylic acid (FDCA) is still scarce. In this context, a series of poly(1,4-butylene 2,5-furandicarboxylate)-*co*-poly(poly(propylene oxide) 2,5-furandicarboxylate) co-polyesters with different composition of stiff poly(1,4-butylene 2,5-furandicarboxylate) (PBF) and soft poly(poly(propylene oxide) 2,5-furandicarboxylate) (PPOF) moieties were synthesized, via a two-step bulk polytransesterification reaction. The molar ratio of PBF/PPOF incorporated was varied (10 to 50 mol%) in order to prepare several novel materials with tuned properties. The materials were characterised in detail through several techniques, namely ATR FTIR, ^1^H and ^13^C NMR, TGA, DSC, DMTA and XRD. Their hydrolytic and enzymatic degradation evaluation was also assessed. These new co-polymers showed either a semi-crystalline nature when higher PBF/PPOF ratios were used, and for approximately equal amounts of PBF and PPOF an amorphous co-polyester was obtained instead.

## 1. Introduction

The massive consumption of fossil-based polymers used on a variety of commodity objects of daily life has prompted, in the last decades, the development of renewable-based alternatives with emphasis on their sustainability. Among the renewable-based polymers, polyesters derived from 2,5-furandicarboxylic acid (FDCA) are some of the most promising. FDCA is a renewable-based aromatic building block monomer that has been widely explored as precursor of several homopolyesters, with very similar properties to those of their non-renewable counterparts, namely terephthalic acid-derivatives (TPA) [1,2].

Some examples of polyesters synthesized from FDCA are poly(ethylene 2,5-furandicarboxylate) (PEF) [3] and poly(1,4-butylene 2,5-furandicarboxylate) (PBF), among others, with similar properties to those of their TPA counterparts [4,5,6,7,8,9,10,11] or even enhanced properties such as for example barrier properties or degradability under enzymatic conditions [12,13]. Furthermore, several other diols besides linear ones have been explored to prepare FDCA-based homopolymers, namely branched diols such as 1,2-propanediol, 2,3-butanediol, 2-methyl-1,3-propanediol and 2,2-dimethyl-1,3-propanediol [14,15,16,17,18,19,20]. The ensuing materials presented even higher *T_g_*s than those of their corresponding homopolyesters synthesized with linear diols with the same number of carbon atoms.

In addition, a demand for new polyesters with specific properties emerged due to the necessity to fulfil specific application gaps, namely in the fields of biomaterials or elastomeric compounds. In this context, some studies [11,21,22,23,24] have been focused on the synthesis of FDCA-based poly(ester-ether)s (PEE’s) co-polymers composed of polyether soft moieties, for example, poly(butylene glycol) (PBG) [24] and/or poly(ethylene glycol) (PEG) [22,23] to replace their fossil-based counterparts, such as poly(butylene 1,4-terephthalate)-*co*-poly(poly(butylene glycol) 1,4-terephtalate) (PBT-*co*-PBGT) or poly(butylene 1,4-terephthalate)-*co*-poly(poly(ethylene glycol) 1,4-terephtalate) (PBT-*co*-PEGT). Commercial PEEs based on TPA, find important applications among the biomedical field [25,26]. Indeed, PBT-*co*-PEGT co-polymers are commercial PEEs (under trade mark of PolyActive®) [27], and are widely used for drug delivery systems, presenting high thermal stability, as well as enhanced flexibility when compared to PET. This class of polymers show great potential, especially if prepared from FDCA, nonetheless, the literature in furanic PEEs is still scarce [11,21,22,23,24]. Zhou et al. [21] presented the first study, reporting the synthesis of poly(1,4-butylene 2,5-furandicarboxylate)-*co*-poly(poly(butylene glycol) 2,5-furandicarboxylate) (PBF-*co*-PBGF) co-polyesters, with enhanced thermal (maximum decomposition temperatures varying from 363 to 378 °C) and mechanical properties (elongation at break between 381 to 832%). Sousa et al. [11] also reported the synthesis of new poly(ester-ether)s co-polymers from FDCA and PEG with different molecular weights (*M_n_* of ~ 200, 400 and 2000), and isosorbide, possessing high thermal stability. More recently, a series of poly(1,4-butylene 2,5-furandicarboxylate)-*co*-poly(poly(ethylene glycol) 2,5-furandicarboxylate) (PBF-*co*-PEGF) co-polyesters were also reported, displaying high thermal stability (up to 380 °C) and low *T_g_*s (ranging from −43.1 to −35.4 °C), enabling their manufacturing process [22,23]. Hydrolytic degradability of PBF-*b*-PEGF was also studied, showing that they can be easily hydrolyzed under alkaline conditions (in phosphate buffered saline (PBS) solution at pH = 12) [23]. Further, Chi et al. [24] also synthesized several PEE’s from FDCA, neopentyl glycol and poly(butylene glycol), with enhanced flexibility (elongation at break values from 38 to 1281%).

However, to our knowledge, the use of poly(propylene oxide) (PPO) as co-monomer for the synthesis of PPE co-polymers has not been reported in the literature before. In this context, co-polymerization of FDCA, 1,4-butanediol (BD) and PPO, could be an elegant way to obtain materials with low *T_g_*, facilitating its processability and, at same time, maintaining the thermal stability, thus enlarging the role of potential applications. Precisely, in this study, a series of poly(1,4-butylene 2,5-furandicarboxylate)-*co*-poly(poly(propylene oxide) 2,5-furandicarboxylate) (PBF-*co*-PPOF) co-polyesters were synthesized for the first time by a typical two-step bulk polytransesterification procedure. The ensuing co-polyesters were extensively characterized by SEC, ATR FTIR, ^1^H and ^13^C NMR, TGA, DSC, and DMTA analyses. Importantly, PBF-*co*-PPOF bearing 20 mol% of PPOF moieties was also submitted to a hydrolytic and enzymatic degradability evaluation.

## 2. Materials and Methods

### 2.1. Materials

1,4-Butanediol (BD, 99%), poly(propylene oxide) (PPO, average *M_n_* ~1000), deuterated chloroform (CDCl_3_-*d*, 99 atom% D), titanium(IV) tert-butoxide (Ti(OBu)_4_, 97%), poly(methyl methacrylate) standards with molecular weights between 4250 and 273,000 sodium phosphate dibasic (≥99%), sodium phosphate monobasic (≥99%) and Porcine pancreas lipase (Type II, 100-500 units/mg protein) were purchased from Sigma-Aldrich Chemicals Co. 2,5-Furandicarboxylic acid (FDCA, >98%) was purchased from TCI Europe NV. Concentrated hydrochloric acid (37%) was purchased from Panreac; and methanol and chloroform (pro-analysis and HPLC grade) were purchased from Fisher Scientific. All chemicals were used as received, without further purification.

### 2.2. Synthesis of Dimethyl 2,5-Furandicarboxylate Monomer

The synthesis of DMFDC followed a previously reported procedure [3]. Typically, DMFDC was prepared by reacting FDCA with an excess of methanol, under acidic conditions (HCl), at 80 °C for 15 h. The final product was isolated in 71% yield as a white powder. FTIR (ν/cm^−1^): 3168 (=C–H); 2965 (C–H); 1706 (C=O); 1578, 1522 (C=C); 1288 (C–O); 1024 (furan ring breathing); 969, 825, 757 (2,5-dibustituted furan ring). ^1^H NMR (300 MHz, CDCl_3_, δ/ ppm): 7.2 (s, *H3*/*H4* furan ring); 3.9 (s, 2,5-COOC*H3*). ^13^C NMR (75 MHz, CDCl_3_, δ/ppm): 158 (2,5-C=O); 147 (*C2*/*C5* furan ring); 118 (*C3*/*C4* furan ring); 52 (2,5-COO*C*H_3_).

### 2.3. Syntheses of Poly(1,4-Butylene 2,5-Furandicarboxylate)-Co-Poly(Poly(Propylene Oxide) Co-Polymers and Poly(1,4-Butylene 2,5-Furandicarboxylate)

The polyesters were prepared by a two-step bulk polytransesterification approach following an adapted procedure reported elsewhere [11]. Reactions were carried out by mixing DMFDC (ca. 0.56 g, 3.04 mmol) and a slight excess amount of BD and PPO (ca. 3.22 mmol) (BD/PPO molar ratios of 100/0, 90/10, 80/20, 50/50, Table 1), in the presence Ti(OBu)_4_ as catalyst (1 wt% relative to the total mass of monomers). In the first step, the mixture was heated progressively from 100 up to 190 °C, for 5 h, under a nitrogen atmosphere and with constant stirring. In the second step, the reaction proceeded under vacuum (10^−3^ bar) and the temperature was slowly raised to 200 °C for 1h, and finally kept at 210 °C, for 2 h. The ensuing solid products were purified by dissolving in chloroform (~20 mL), and then pouring in an excess of cold methanol, filtered, dried and weighted. In the case of PBF-*co*-PPOF 50/50 co-polyester (BD/PPO = 50/50), viscous liquid at room temperature, a liquid-liquid extraction procedure using chloroform (~20 mL) was used instead.

Hereafter, the co-polyesters will be referred to as PBF-*co*-PPOF 90/10, 80/20 and 50/50, according to the BD/PPO molar ratio used as feed. Table 1 presents the molar amounts of each monomer used as well as the weight average molecular weights (*M*_w_) and dispersity (*Đ* = *M*_w_/*M*_n_) of the polymers.

### 2.4. Characterization Methods

Size-exclusion chromatography (SEC) analyses of co-polyesters were performed on a Viscotek (Viscotek TDAmax) (Malvern, Gondomar, Portugal) equipped with a differential viscometer (DV) and right-angle laser-light scattering (RALLS, Viscotek) and refractive index (RI) detectors. The column set consisted of a PLgel 5 μm guard column followed by two columns, namely Viscotek T5000 and T4000 column, respectively. A dual piston pump was set with a flow rate of 1 mL min^−1^. The eluent (DMF with 0.03% LiBr) was previously filtered through a 0.2 μm filter. The system was also equipped with an on-line degasser. The analyses were performed at 60 °C using an Elder CH-150 heater. Before injection, the samples were filtered through a PTFE membrane with 0.2 μm pore. The system was calibrated with narrow poly(methyl methacrylate) standards and the molecular weight and dispersity of the polymers were determined by conventional calibration.

Attenuated total reflectance Fourier transform infrared (ATR FTIR) spectra were obtained using a PARAGON 1000 FTIR spectrometer equipped with a single-horizontal Golden Gate ATR cell (Perkin-Elmer, MA, United States). The spectra were recorded after 128 scans, at a resolution of 4 cm^−1^, within the range of 500 to 4000 cm^−1^.

^1^H, ^13^C nuclear magnetic resonance (NMR) spectra were recorded in CDCl_3_ using a Bruker AMX 300 spectrometer (Bruker, Madrid, Spain), operating at 300 or 75 MHz, respectively. All chemical shifts (δ) are expressed as parts per million (ppm), downfield from tetramethylsilane (used as the internal standard). Further, the calculation of the real incorporation of BD/PPO ratio (PBF/PPOF_real_) the integration areas of OC*H_2_* proton resonance of F-BD diad (δ ≈ 4.40 ppm) and of F-PPO diad (δ ≈ 3.93 ppm) were used, according to the equation: $\left[A_{\mathrm{OCH}2;F-\mathrm{BD}}/\left(A_{\mathrm{OC}H2;F-\mathrm{BD}}+A_{\mathrm{OC}H2;F-\mathrm{PPO}}\right)]/[A_{\mathrm{OC}H2;F-\mathrm{PPO}}/\left(A_{\mathrm{OC}H2;F-\mathrm{DB}}+A_{\mathrm{OC}H2;F-\mathrm{PPO}}\right)\right]$.

The Average PBF sequence length was also calculated by the equation: $L_{n,BF}=1/\left[\left(1-\left(PBF_{real}/100\right)\right)\right]$ [28].

Thermogravimetric analyses (TGA) were carried out with a Setaram SETSYS analyzer (Setaram, Caluire, France) equipped with an alumina plate. Thermograms were recorded under a nitrogen flow of 20 mL min^−1^ and heated at a constant rate of 10 °C min^−1^ from room temperature up to 800 °C. Thermal decomposition temperatures were taken at 5% weight loss step and at maximum decomposition temperatures from the heated samples (*T*_*d*,5%_ and *T_d,max_*, respectively).

Differential scanning calorimetry (DSC) thermograms were obtained with a Hitachi DSC7000X calorimeter (Hitachi, Paris, France) equipped with a liquid nitrogen cooling system, using aluminum DSC pans. Scans were carried out under nitrogen with a heating rate of 5 °C min^−1^ in the temperature range from −90 to 200 °C. Two heating/cooling cycles were repeated. Glass transition temperature (*T_g_*) was determined using the midpoint approach (second heating trace); and cold crystallization (*T_cc_*) and melting (*T_m_*) temperatures were determined as the maximum of the exothermic crystallization peak and the minimum of the melting endothermic peak during the second heating scan, respectively.

Dynamic mechanical thermal analyses (DMTA) were performed using a material pocket accessory with a Tritec 2000 DMA (Triton, WA, United States), operating in the single cantilever mode. Tests were performed at 1 and 10 Hz and the temperature was varied from -100 to 200 °C, at 2 °C min^−1^. *T_g_* was determined as the maximum peak of tan δ.

X-ray diffraction (XRD) measurements were performed using a Philips X’pert MPD diffractometer operating with CuKα radiation (λ = 1.5405980 Å) (Malvern, Gondomar, Portugal) at 40 kV and 50 mA. Samples were scanned in the 2*θ* range of 5 to 50°, with a step size of 0.04°, and time per step of 50 s.

In vitro hydrolytic and enzymatic degradation tests were carried out using press-molded square-shape samples (ca. 69–113 mg) of the prepared polyesters and placed into closed containers with phosphate buffer saline solution (PBS) (10 mL) or with a PBS solution (10 mL) containing Porcine pancreas lipase (concentration of 0.1 mg mL^−1^), for each test, respectively. The specimens were taken out of the related solution at regular intervals (each 7 days), rinsed thoroughly with distilled water, dried at room temperature for 4 h and, weighed. To prevent saturation, both solutions were renewed every 7 days. Each measurement was repeated five times. The weight loss percentage was calculated using the expression: $\mathrm{Weight}\;\mathrm{loss}\;\left(\%\right)=\left[\left(W_{0}-W_{d}\right)/W_{0}\right]\times 100$, where, W_o_ and W_d_ stand for the specimens’ weights prior and after incubation, respectively.

## 3. Results and Discussion

### 3.1. PBF-co-PPOF Co-Polyesters Synthesis and Structural Characterization

**Scheme 1:**
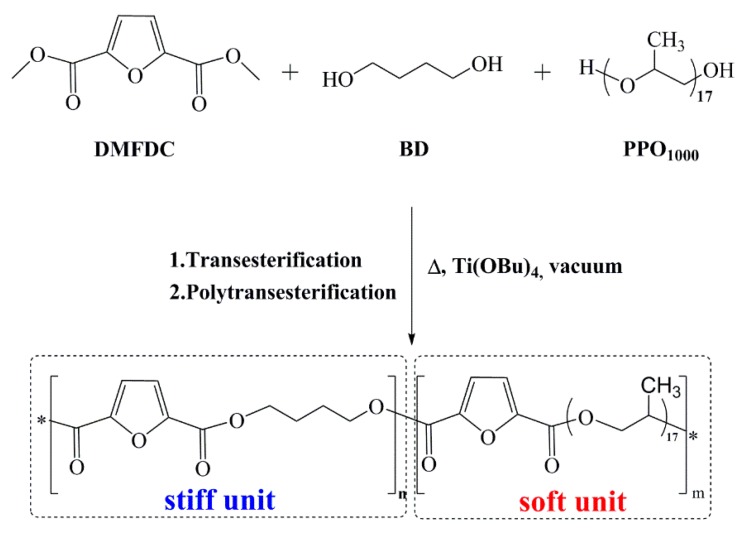
Synthesis of PBF-*co*-PPOF co-polymers.

In this study the newly prepared poly(ester-ether) co-polymers are based on poly(1,4-butylene 2,5-furandicarboxylate) as rigid unit and on a soft segment derived from poly(propylene oxide) (Scheme 1). Interestingly, the poly(ether) selected has a methyl side group which plays an important role on the structure-properties features, as discussed ahead.

PBF-*co*-PPOF were synthesized via a two-step conventional melt polytransesterification approach (Scheme 1) [3], in the presence of Ti(OBu)_4_ catalyst and at relatively moderate temperatures, not exceeding 210 °C, to avoid undesirable side reactions involving the furan moiety (e.g., decarboxylation reactions which are commonly associated to color problem issues) [1]. The resulting polymers were isolated as powders (PBF, PBF-*co*-PPOF-90/10 and 80/20) or a viscous liquid (PBF-*co*-PPOF-50/50), in relatively good yields ranging from 65 to 71% (Table 1) in similarity to other FDCA-based polyesters [22]. Furthermore, these co-polyesters showed weight-average molecular weight (*M_w_*) values between 36,700–48,500, and dispersity (*Đ*) around 2.

**Figure 1:**
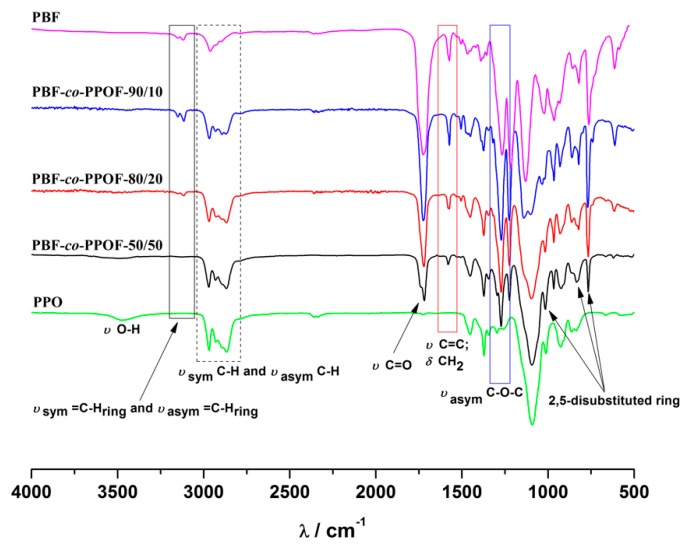
ATR FTIR spectra of PBF-*co*-PPOF co-polymers.

Typical ATR FTIR spectra of all PBF-*co*-PPOF co-polyesters and related PBF homopolyester (Figure 1) displayed two weak bands near 3150 and 3115 cm^−1^ attributed to the ν C-H bond of the furanic ring. In addition, near 2968, 2930, 2893 and 2868 cm^−1^ there are four weak bands attributed to the anti-symmetrical and symmetrical stretching modes (ν C–H *asym* and ν C–H *sym*, respectively) of the C–H bond of methylene and methyl groups related to the BD and PPO moieties, respectively. Additionally, both PBF and co-polyesters spectra exhibited a very intense band near 1725 cm^−1^, arising from the C–O stretching vibration, typical of ester groups. Two bands at 1506 and 1573 cm^−1^, arising from the C–C bond of the furan ring, and C–O–C stretching vibrations appeared at around 1271 cm^−1^ and the typical vibration modes of 2,5-disubstituted furans were observed at 966, 822, and 769 cm^−1^ in the case of PBF and PBF-*co*-PPOF materials. The presence of the abovementioned bands confirmed the success of the polymerization reactions.

**Figure 2:**
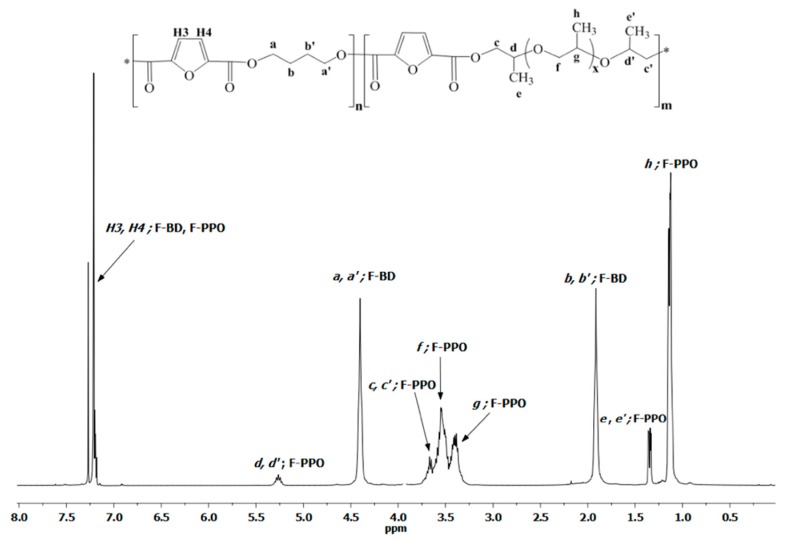
^1^H NMR spectrum of PBF-*co*-PPOF-90/10 co-polymer in CHCl_3_-*d*.

The chemical structure characterization of all PBF-*co*-PPOF co-polyesters and PBF homopolyester was also studied by ^1^H (Figure 2 and Table 2), ^13^C NMR (Table 4 and Figure S1) and 2D analysis (Figure S2). The main ^1^H NMR resonances and respective assignments of all polymers studied are summarized in Table 2 and Figure 2 displays the ^1^H NMR spectrum of PBF-*co*-PPOF-90/10.

The ^1^H NMR spectra of all polymers (Figure 2 and Table 2) displayed the typical resonances attributed to the F-BD diad at approximately *δ* 7.21, 4.40 and 1.91 attributed to the *H3* and *H4* protons of the furan ring, and to the C(O)OC***H_2_***CH_2_ and C(O)OCH_2_C***H_2_*** protons of the BD moiety, respectively. In the co-polymers spectra, the corresponding resonances associated to the PPO-F diads were also detected: *δ* 5.26, 3.67 and 1.34 ppm, arising from the C(O)OC***H***, C(O)OC***H_2_*** and C(O)OCHC***H_3_*** protons, in the neighboring of the furan ring, respectively. Moreover, the protons related to the PPO-PPO units were also identified at *δ* 3.54, 3.40, and 1.15 ppm, related to OC***H_2_*** (ether linkage), OC***H*** and OCHC***H_3_***, respectively.

Furthermore, the ^1^H NMR spectra data was used to access the real molar percentage of PBF and PPOF moieties in the co-polyesters backbone, due to the important impact this ratio has on the ensuing co-polyesters properties. The PBF/PPOF real incorporation was determined using the integration areas of C(O)OC***H_2_*** proton resonances (in the neighboring of furan ring) of the F-BD (*δ* at 4.40 ppm) and F-PPO (*δ* at 3.93 ppm) diads, respectively, and the main results are presented in Table 3.

From Table 3, it is possible to observe that despite the BD and PPO feed ratio, there was a tendency for a higher incorporation of PPOF into co-polyesters chains, most probably associated with BD lost during the polytransesterification step due to the high BD volatility. However, this trend is almost negligible in the case of the PBF-*co*-PPOF-90/10 and 80/20 co-polyesters.

The number average sequence length of BF unit (*L_n,BF_*) was also assessed assuming that PBF-*co*-PPOF co-polyesters are random co-polyesters. It was found that *L_n,BF_* increased with the BF content increasing, according with the theoretically expected values [11,22,29,30]. Importantly, the co-polyester with the highest amount of PBF (PBF-*co*-PPOF-90/10) had a *L_n,BF_* of 6.5. This is an important structural feature that is in accordance with a crystalline domain dominated by PBF segments and corresponding melting behavior, as discussed above.

In terms of ^13^C NMR analysis (Table 4 and Figure S1), the observed resonances were in agreement with their expected chemical structure and corroborated the above ^1^H NMR results, as well as the ATR FTIR data.

### 3.2. X-Ray Diffraction Analysis

**Figure 3:**
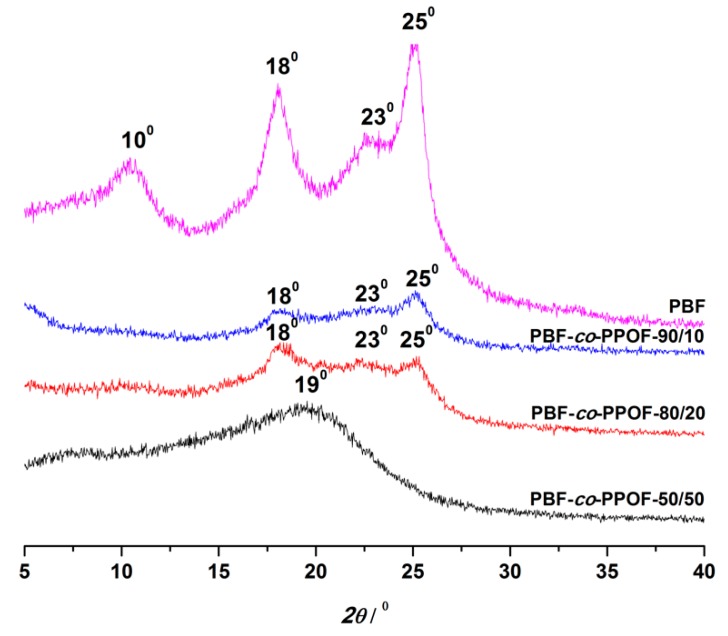
XRD patterns of PBF and all PBF-*co*-PPOF materials.

The XRD patterns of PBF-*co*-PPOF-90/10 and 80/20 co-polymers (Figure 3) indicated that they were semi-crystalline polymers, displaying both an amorphous halo around 2*θ* ~ 21° and diffraction peaks at 2*θ* ≈ 18, 23 and 25°, quite similar to the PBF pattern although in the former case peaks were more intense than for the co-polymers probably due to a higher crystallinity. In fact, the PBF XRD pattern (Figure 3), exhibited intense diffraction peaks at 2*θ* ~ 10, 18, 23 and 25° [16,31,32,33]. This clearly indicates that the ability of PBF-*co*-PPOF co-polyesters to crystallize is mainly associated to PBF segments (with *L_n,BF_* equal to 6.5 and 4.2, respectively). Moreover, these results are in perfect agreement with the below DSC and DMTA data, and also with other FDCA or TPA-based PPEs reported in the literature [21,22,34,35].

As expected, in the case of the viscous liquid PBF-*co*-PPOF-50/50 co-polyester, only a halo centered at 2*θ* ≈ 19 ° was observed, in accordance with an essential amorphous nature.

### 3.3. Thermal Behaviur

PBF-*c*o-PBDG co-polyesters were extensively characterized in terms of their thermal behavior through DSC, DMTA and TGA analyses (Table 5, and Figures S3–S6).

The semi-crystalline character of the co-polymers having higher PBF/PPOF molar ratios (PBF-*co*-PPOF-90/10 and 80/20) was confirmed by DSC and DMTA analyses (Figures S3–S5). For example, the DSC trace of PBF-*co*-PPOF-90/10 co-polyester (Figure S4) displayed glass transition (*T_g_*) and melting temperatures (*T_m_*) at −49.7 and 137.7 °C, respectively. The PBF-*co*-PPOF-80/20 co-polyester also maintained some ability to crystallize and melt, with a cold transition (*T_cc_*) and *T_m_* of approximately 21.7 and 124.1 °C (Table 5), similar to the one of PBF (*T_m_* of 170.0 °C). Furthermore, using DTMA analysis (Figure S5), *T_g_* values of −32.6, −37.2 and −42.3 °C for PBF-*co*-PPOF-90/10, 80/20 and 50/50 materials were observed, respectively; and *T_m_* of 147.6 and 124.2 °C for PBF-*co*-PPOF-90/10 and 80/20 were detected. In the case of PBF-*co*-PPOF-50/50 co-polymer (having a lower PBF/PPOF molar ratio) the absence of *T_m_* is in accordance with its essential amorphous nature (XRD data).

In summary, the highly desirable properties obtained included, the *T_g_* of the co-polyesters decreased with the increasing content of soft PPOF segments (tailored behavior), although these results were still higher than those of amorphous PPO homopolyester (*T_g_* between −67 to −72 °C [36,37]). These results were expected since the incorporation of more PPO flexible moieties into FDCA-based co-polyesters typically gives rise to a decrease on the co-polymers’ thermal features [21,22]. Importantly, the co-polymers with higher BF content, PBF-*co*-PPOF-90/10 and 80/20, showed a typical segmented polymers behavior [22], with a *T_m_* very close to that of stiff PBF and a *T_g_* below room temperature closer to that of soft PPO, revealing that PBF units were the main responsible for the crystalline behavior of the ensuing materials, whereas the PPOF segments were associated with the amorphous domain. Hence, for PBF-*co*-PPOF-90/10 the range of working temperatures was quite enlarged: within −39 and 139 °C.

In general the TGA thermograms (Table 5 and Figure S6) of the co-polyesters (carried out under nitrogen atmosphere) exhibited one major characteristic event at the maximum decomposition temperatures (*T_d,max_*) of 340−365 °C. Also, the newly prepared co-polymers showed to be thermally stable up to *T*_*d*,5%_ ≈ 308 °C.

As shown in Table 5, the co-polymers had both *T*_*d*,5%_ and *T_d,max_* results lower than those observed to PBF. These less favorable thermal results could be associated with the presence of the appending methyl group, as already reported for other polyesters also having side groups, such as poly(2,3-butylene 2,5-furandicarboxylate) compared to PBF [17]. Nevertheless, all PBF-*co*-PPOF co-polyesters have higher *T_d,max_* than PPO.

### 3.4. Hydrolytic and Enzymatic Degradation Tests

**Figure 4:**
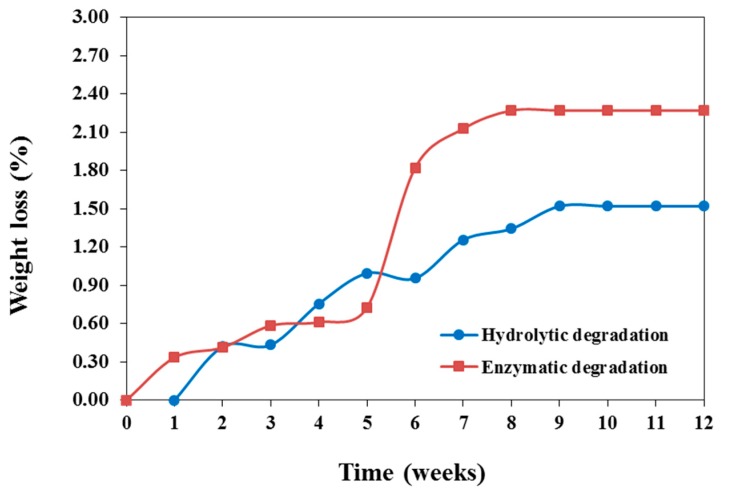
Weight loss percentage of PBF-*co*-PPOF-80/20 co-polyester along 12 weeks.

As mentioned above, in addition to specific thermal properties, it was also important to understand if the newly prepared materials degraded under hydrolytic and enzymatic conditions for a relatively short period of time. The evaluation of the hydrolytic and enzymatic degradation behavior was performed in terms of weight loss percentage versus time (Figure 4) for PBF-*co*-PPOF-80/20 co-polyester, with real incorporation of PPOF moieties around 24 mol%. However, the polymer’s weight loss under hydrolytic conditions was almost negligible for a relatively short period of 12 weeks, and a little bit higher under enzymatic conditions (2.3%). This result was in the same line with those reported for PBF-*co*-PEGF co-polyesters incorporating similar amount of PBF moieties [23], and also with studies on PBT-*co*-PEGT co-polyesters in similar hydrolytic conditions as the ones used in this study (pH ~ 7 and 37 °C) [38]. 

The incubation with a Porcine pancreas lipase in PBS solution slightly increased the weight loss of the co-polyester, but it was still very low. Through hydrolytic and enzymatic degradation only 1.5 and 2.3% weight loss were achieved, respectively. Nevertheless, the data obtained are good preliminary results, and showed some evidence of degradation of PBF-*co*-PPOF (also checked by FTIR) associated with the hydrolysis of the ester groups, but further studies using other enzymes such as for example cutinases, could enhance the results obtained. Some positive results were previously obtained with cutinase from *Humicola insolens* or *Thermobifida cellulosilytica* to hydrolyze PET and PEF [12,13]. Further, incorporation of PPO in the co-polymers, is also, expected to enhance degradability.

## 5. Conclusions

In conclusion, a new class of FDCA-based poly(ester-ether) co-polymers has been accomplished, incorporating both stiff and soft moieties into their backbone. The ensuing co-polyesters have shown high thermal stability (*T_d,max_* between 340 to 365 °C) and *T_g_* at sub-ambient temperatures, namely from −42.3 to −32.6 °C. Moreover, the semi-crystalline character was only observed for co-polyesters with higher BF content, revealing that PBF units were mainly responsible for the crystalline behavior of the ensuing materials, whereas the PPOF were associated with the amorphous domain. Furthermore, PBF-*co*-PPOF-80/20 co-polyester had shown a week hydrolysable behavior, presenting a maximum percentage weight loss of 2.3 %, after 12 weeks.

Finally, due to their high thermal stability, as well as the presence of both stiff and soft moieties in the co-polymer chains, these materials could find interesting industrial applications, namely as thermoplastic polymers.

## Figures and Tables

**Table 1:** Molecular characteristics of PBF and PBF-*co*-PPOF *co*-polymers.

(co)polymer	BD/PPO_feed_^1^ (mol%)	Yield (%) ^2^	*M_w_* ^3^	*Đ* ^4^
PBF	100/0	71.0	-	- ^5^
PBF-*co*-PPOF-90/10	90/10	64.8	36 700	2.2
PBF-*co*-PPOF-80/20	80/20	68.1	41 600	2.2
PBF-*co*-PPOF-50/50	50/50	71.4	48 500	2.1

^1^ Molar feed percentage of BD and PPO units. ^2^ Isolation yields of purified polyesters in methanol. ^3^ Weight-average molecular weight. ^4^ Dispersity. ^5^ Not determinate due to the insolubility of PBF in DMF.

**Table 2:** Main ^1^H NMR resonances of PBF-*co*-PPOF co-polyesters, and PBF and PPO homopolyesters. The a, b, c, d, e, f, g and a′, b′, c′, d′, e′ attributions are explained in the scheme of Figure 2.

δ/ppm	Assignment	Diads	Integration Area				
			PBF	PBF-*co*-PPOF			PPO
				90/10	80/20	50/50	
7.21	***H3*** and ***H4***; C***H***	F-BD; FDCA-PPO	1.00	1.00	1.00	1.00	
5.26	***d***, ***d′***; C***H***CH_3_	F-PPO	–	0.14	0.37	0.92	
4.40	***a***, ***a′***; C(O)OC*H_2_*CH_2_	F-BD	2.01	1.66	1.28	0.46	
3.67	***c***, ***c′***; C(O)OC***H_2_***	F-PPO	–	0.30	0.84	1.84	
3.54	***f***; OC***H_2_***	PPO-PPO	–	1.91	5.14	17.89	2.01
3.40	***g***; OC***H***	PPO-PPO	–	1.03	2.77	9.46	1.00
1.91	***b***, ***b′***; C(O)OCH_2_C***H_2_***	F-BD	2.01	1.66	1.41	0.48	
1.34	***e***, ***e′***; CHC***H_3_***	F-PPO		0.44	1.30	3.04	
1.15	***h***; OCHC***H_3_***	PPO-PPO	–	3.10	8.60	28.50	3.02

**Table 3:** Comparison between the initial and the real molar ratio percentages of PBF and PPOF in the polyesters’ backbone.

(co)polymer	PBF/PPOF_feed_^1^	PBF/PPOF_real_^2^	*L_n,BF_* ^3^
PBF	100/0	100/0	–
PBF-*co*-PPOF-90/10	90/10	85/15	6.5
PBF-*co*-PPOF-80/20	80/20	76/24	4.2
PBF-*co*-PPPOF-50/50	50/50	20/80	1.3

^1^ Molar feed ratio percentage of BD and PPO units. ^2^ Molar real ratio percentage of BD and PPO units. ^3^ Average PBF sequence length.

**Table 4:** Main ^13^C NMR resonances of all PBF-*co*-PPOF co-polyesters, and PBF and PPO homopolyester. The a, b, c, d, e, f, g and a′, b′, c′, d′, e′ attributions are explained in the scheme of Figure 2.

Assignment	Diads	Integration Area				
		PBF	PBF-*co*-PPOF			PPO
			90/10	80/20	50/50	
**2-*C***O and ***5-C***O; ***C***(O)O	F-BD; F-PPO	158.0	158.0	158.0	158.0	-
***C2*** and ***C5***; ***C***-C(O)O	F-BD; F-PPO	146.8	146.8	146.8	147.0	-
***C3*** and ***C4***; ***C***-H	F-BD; F-PPO	118.5	118.5	118.5	118.2	-
***f***; O***C***H_2_	PPO-PPO	-	75.4	75.3	75.3	75.3
***g***; O***C***H	PPO-PPO	-	73.4	73.4	73.4	74.4
***c***, ***c′***; C(O)O***C***H_2_	F-PPO	-	72.9	72.9	72.9	-
***d***, ***d′***; ***C***HCH_3_	F-PPO	-	71.7	71.7	71.3	-
***a***, ***a′***; C(O)O***C***H_2_CH_2_	F-BD	64.9	64.9	64.9	64.8	-
***b***, ***b′***; C(O)OCH_2_***C***H_2_	F-BD	25.4	25.4	25.3	25.0	-
***h*** ; OCH***C***H_3_	PPO-PPO	-	17.3	17.3	17.3	17.3
***e***, ***e′***; CH***C***H_3_	F-PPO	-	16.9	16.8	16.8	-

**Table 5:** Decomposition at 5% weight loss (*T*_*d*,5%_), maximum decomposition (*T_d,max_*), glass transition (*T_g_*) and melting temperature (*T_m_*) of PBF, PPO and PBF-*co*-PPOF co-polyesters.

co(polymer)	DSC ^1^	DMTA ^2^		TGA ^3^	
	*T_m_*/°C	*T_g_*/°C	*T_m_*/°C	*T_d_*_,5%_/°C	*T_d,max_*/°C
PBF	170.0	75.6	166.9	348.7	380.5
PBF-*co*-PPOF-90/10	137.7	−32.6	147.6	284.7	347.2
PBF-*co*-PPOF-80/20	124.1	−37.2	124.2	288.9	340.3
PBF-*co*-PPOF-50/50	-	−42.3	- ^3^	308.1	365.2
PPO		−67.0–72.0 ^5^	-	283.0	323.4

^1^ Determined by DSC analysis. ^2^ Determined by DMTA analysis. ^3^ Determined by TGA analysis. ^4^ Values obtained from references [36,37].

## Supplementary Materials

The following are available online at http://www.mdpi.com/1996-1944/12/2/328/s1, Figure S1: ^13^C NMR spectra in CHCl_3_-d of PBF-*co*-PPOF-90/10 copolymer, Figure S2: 2D NMR spectrum in CHCl_3_-d of PBF-*co*-PPOF-90/10 copolymer, Figure S3: DSC curve of PBF homopolyester. Figure S4: DSC curves (second scan) of all PBF-*co*-PPOF copolymers studied, Figure S5: Tan δ of PBF and PBF-*co*-PPOF (co)polymers, at 1 Hz, Figure S6: TGA (a) and DTGA (b) thermograms of PBF-*co*-PPOF copolymers and PBF homopolyester.
