# Supplementary material for: Co-Polymers based on Poly(1,4-butylene 2,5-furandicarboxylate) and Poly(propylene oxide) with Tuneable Thermal Properties: Synthesis and Characterization
Source: Materials (Basel). 2019 Jan 21;12(2):328. doi: 10.3390/ma12020328 (PMC6356699; doi:10.3390/ma12020328)
Supplement: Supplementary file 1 [file materials-12-00328-s001.pdf]

materials

Supplementary Materials

Copolymers based on poly(1,4-butylene 2,5-furandicarboxylate) and Poly(Propylene Oxide) with Tuneable Thermal Properties: Synthesis and Characterization

1. Structural Characterization

1.1. NMR Results

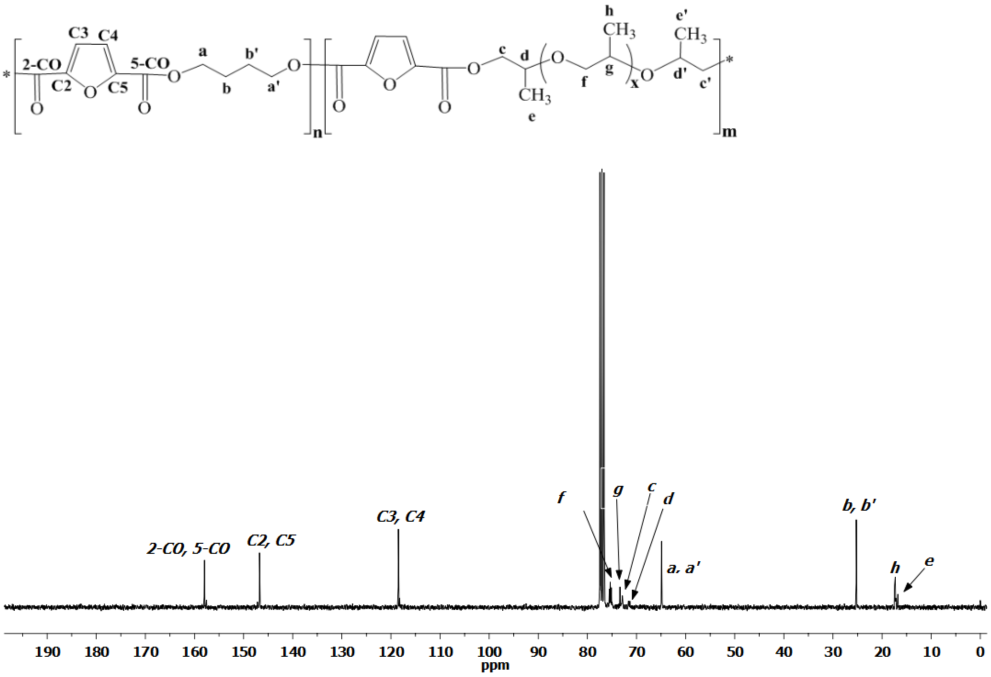

Figure S1. ^{13}C NMR spectra in $CHCl_3$-*d* of PBF-*co*-PPOF-90/10 copolymer.

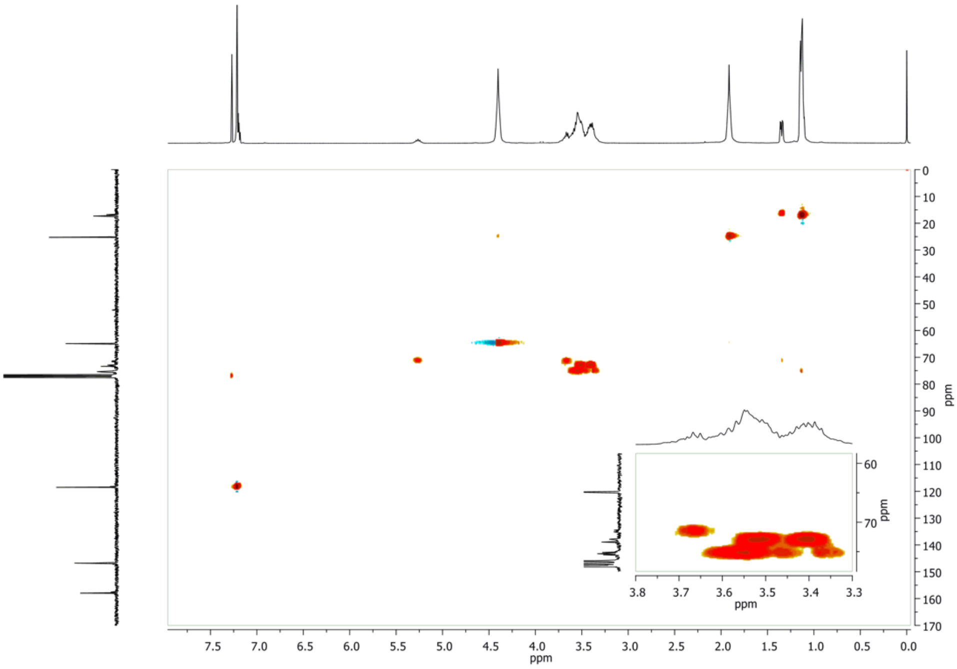

Figure S2. 2D NMR spectrum in $CHCl_3$-*d* of PBF-*co*-PPOF-90/10 copolymer.

1.2. Thermal Behaviour

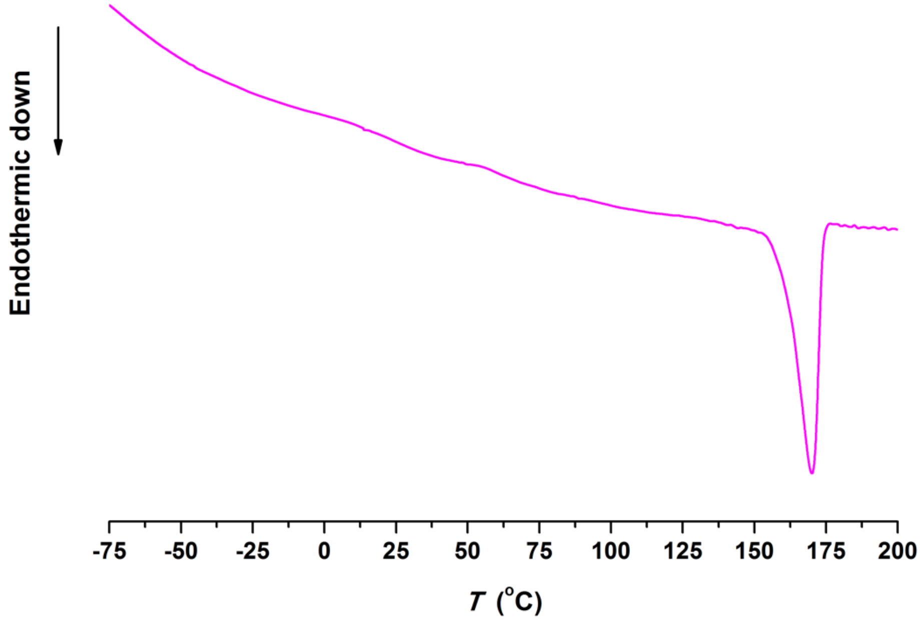

Figure S3. DSC curve of PBF homopolyester.

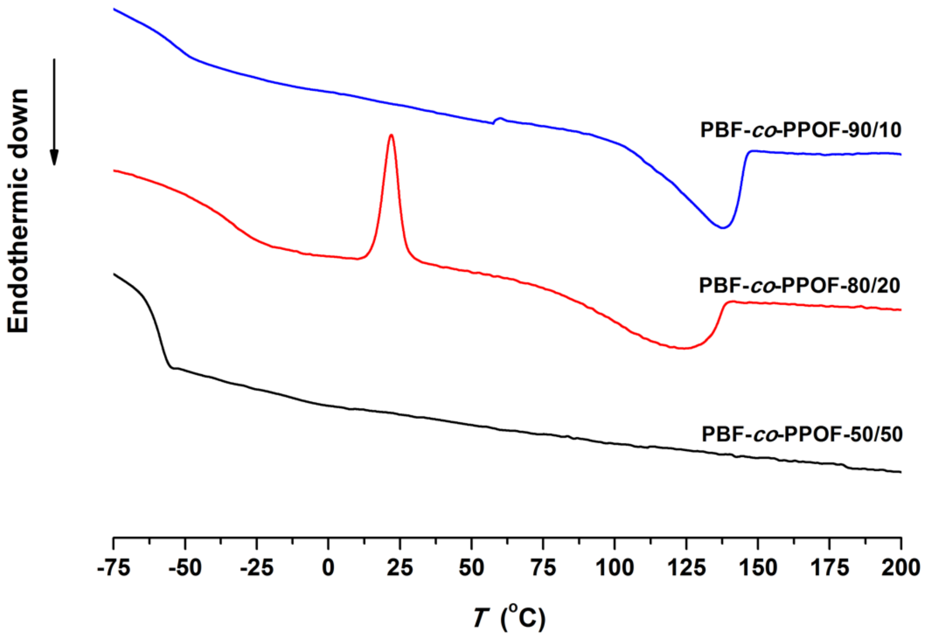

Figure S4. DSC curves (second scan) of all PBF-co-PPOF copolymers studied.

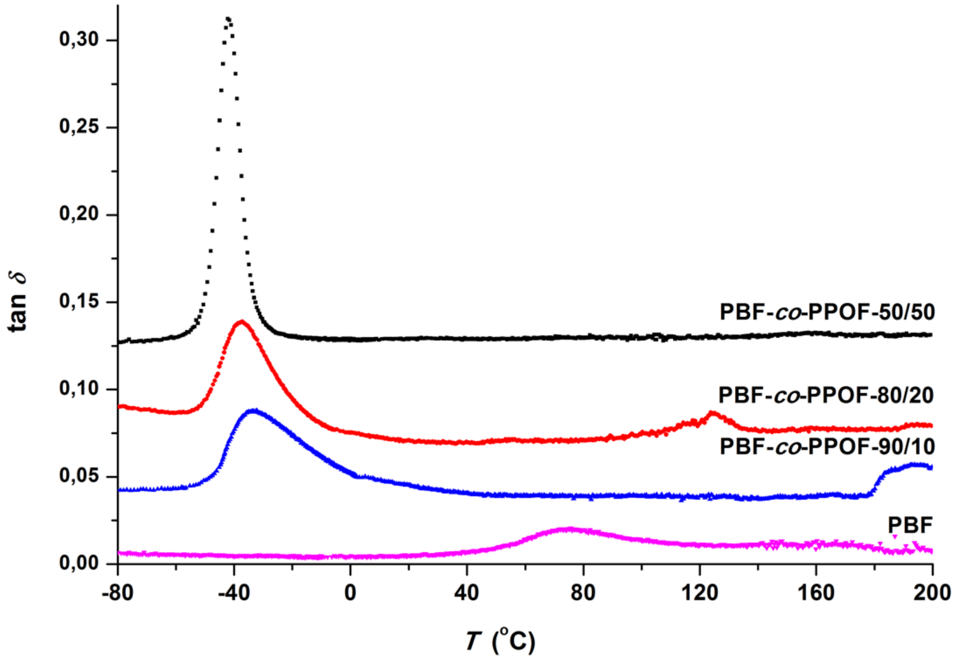

Figure S5. Tan δ of PBF and PBF-co-PPOF (co)polymers, at 1 Hz.

materials

MDPI

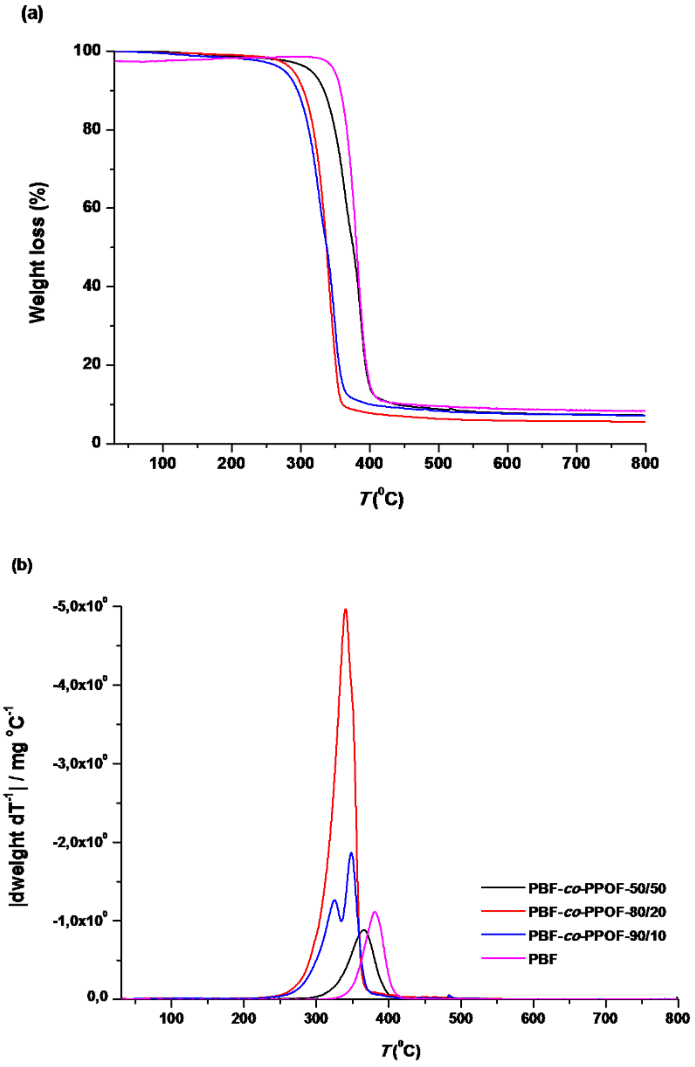

Figure S6. TGA (a) and DTGA (b) thermograms of PBF-co-PPOF copolymers and PBF homopolyester.
